# Maintenance chemotherapy using S-1 following definitive chemoradiotherapy in patients with N3 nasopharyngeal carcinoma

**DOI:** 10.1186/s13014-019-1387-9

**Published:** 2019-10-22

**Authors:** Jingfeng Zong, Hanchuan Xu, Bijuan Chen, Qiaojuan Guo, Yun Xu, Chuanben Chen, Youliang Weng, Wei Zheng, Jianji Pan, Shaojun Lin

**Affiliations:** Department of Radiation Oncology, Fujian Cancer Hospital & Fujian Medical University Cancer Hospital, No. 420 Fuma Road, Fuzhou, 350014 China

**Keywords:** Maintenance chemotherapy, N3, Nasopharyngeal carcinoma, Chemoradiotherapy, S-1

## Abstract

**Background:**

Patients with N3 stage nasopharyngeal carcinoma (NPC) are at high risk for treatment failure. This study aims to assess the efficacy of maintenance chemotherapy (MC) using S-1 (MC-S1), a novel oral fluoropyrimidine agent, following definitive chemoradiotherapy (CRT) using intensity-modulated radiotherapy (IMRT) in patients with N3 nasopharyngeal carcinoma (N3-NPC).

**Methods:**

A retrospective review was conducted for all N3-NPC treated with CRT with MC (CRT-MC) or without MC (CRT-non-MC) during 2014–2016. Toxicities with MC were recorded. Overall survival (OS), locoregional failure-free survival (LFFS) and distant metastasis free survival (DMFS) were compared between CRT-MC vs. CRT-non-MC cohorts.

**Results:**

A total of 130 N3 patients were identified, of whom 21 (16.2%) were treated with CRT-MC, and 109 (83.8%) with CRT-non-MC. Patient characteristics did not significantly differ between the CRT-MC and CRT-non-MC groups, with the exception of the number of cycles of neoadjuvant chemotherapy. Following IMRT 69 patients achieved a complete response (CR) (CRT-MC: 10; CRT-non-MC: 59), 61 had a partial response (PR) (11 vs. 50), and none maintained stable disease (SD) or developed progression of disease (PD). After a median follow-up of 41 months for surviving patients, a significant differences in OS (76.3% vs. 95.2%, *p* = 0.046) and DMFS (70.3% vs. 90.5%, *p* = 0.043) but not LFFS (84.9% vs. 100%, *p* = 0.091) at 3 years were observed between the CRT-non-MC and CRT-MC groups. Skin hyperpigmentation, leucopenia, fatigue, neutropenia, anorexia and nausea were the common but not severe (grade 1–2) toxicities of MC.

**Conclusions:**

Using MC-S1 in N3-NPC patients following IMRT achieved superior survival to the CRT-non-MC patients. The toxicities of MC-S1 were mild and tolerable. Further clinical trials are required to evaluate the efficacy of MC-S1 in N3-NPC patients.

## Background

Nasopharyngeal carcinoma (NPC) is an endemic malignancy in South China that is highly sensitive to both radiotherapy (RT) and chemotherapy [1]. Due to its being asymptomatic in early stages and prone to lymph node metastasis,about 70% of NPC presents as local advanced disease or regional lymph node metastasis, and definitive concurrent chemoradiotherapy (CRT) is recommended as the standard of care [1]. The overall survival (OS) rate of advanced NPC patients treated with CRT is high, up to 80% at 5-years [2]. However, the 8-year OS rate decreases steeply to about 50% when patients were originally diagnosed with N3 NPC (N3-NPC); defined as cervical lymph nodes larger than 6 cm and/or extension below the caudal border of the cricoid cartilage (regardless of laterality) and are considered to have the highest metastatic risk [3].

Despite of the fact that several high level studies had verified that compared to concurrent chemoradiotherapy (CCRT) alone, adjuvant chemotherapy (AC) following CCRT (CCRT-AC), which is hypothesized to eradicate micrometastases, had not achieved survival benefits in advanced NPC [1]; although it had been thought to benefit survival of N3-NPC patients [4, 5]. However, intensive AC should be conducted cautiously due to potential patient intolerance to chemotherapy that can result from the acute toxicity of CCRT [6]. Thus, CCRT that followed neoadjuvant chemotherapy (NAC-CCRT) rather than CCRT-AC was thought to be a more feasible and effective strategy of treatment [7]. However, even treated with NAC combined with concurrent chemotherapy (CC), distant metastasis (DM) continuous to be the main cause of treatment failure [7]. Therefore, developing novel chemotherapy strategies to decrease DM is the foremost management objective to improve survival of N3-NPC patients.

Maintenance chemotherapy (MC), one of the chemotherapy strategies that includes continuation and switch maintenance, aims to prolong the duration of the response and maintain a quality of life that generally uses a well-tolerated, single-agent regimen of chemotherapy as continued therapy [8]. MC was originated for hematologic malignancies and applied subsequently for several metastatic solid tumors, such as lung and colorectal cancer [8–10]. MC has been reported to achieve a satisfactory high response rate and superior survival in NPC patients with distant metastasis (DM) as well [11, 12]. However, its efficacy in a curative setting has rarely been reported. To the best of our knowledge, the efficacy of MC after definitive CRT in N3-NPC patients, who were prone to develop DM and hypothesized to benefit from MC, has not been reported.

The drug S-1 is a novel oral fluoropyrimidine derivative consisting of tegafur (FT), 5-chloro-2, 4-dihydroxypyridine (CDHP), and potassium oxonate (Oxo), mixed at the molar ratio of 1:0.4:1. It is designed to enhance the clinical utility of an oral fluoropyrimidineand is associated with low gastrointestinal toxicity [13]. Several researches have demonstrated that S-1 alone or in combination with other chemotherapy drugs yielded an excellent survival benefit in NPC patients as first or second line of chemotherapy [5, 14, 15]. Furthermore, S-1 is considered an appropriate regimen drug for MC owing to its convenience for outpatient oral administration, tolerable toxicity, favorable antitumor activity and improved survival [16, 17]. Based on these reports, we have recommend that N3-NPC patients proceed to maintenance chemotherapy using S-1 (MC-S1) following definitive CRT using intensity modulated radiation therapy (IMRT) technology in our center since 2014. This study reviews our experience with MC-S1 following CRT in N3-NPC patients.

## Methods

### Patient selection criteria

This retrospective study was approved by the Ethics Committee of Fujian Cancer Hospital (No.200908). All patients provided written informed consent prior to treatment, and all information was anonymized prior to analysis.

The eligibility and exclusion criteria for the present retrospective study were as follows: histologically proven T any N3 M0 (by 8th edition UICC/AJCC TNM) NPC, age between 18 and 70 years, good performance status (Eastern Cooperative Oncology Group performance status score ≤ 1), adequate organ function (bone marrow, hepatic, and renal) for chemotherapy, initially treated with definitive chemo-radiotherapy (neoadjuvant +/− concurrent chemotherapy) with IMRT, and sufficient follow-up data available for short-term treatment response and survival assessment.

### Treatment

All patients were initially treated with at least two cycles of neoadjuvant (NAC) followed by definitive IMRT alone or Chemo-IMRT with platinum-based agents. After IMRT, adjuvant chemotherapy (AC) and MC were administrated depending on the patients’ preference. AC regiments were dependent on the short-term response to IMRT: the same chemotherapy regimen as NAC if there was a complete response (CR) or partial response (PR), or a different regimen if there was stable disease (SD) or progression of disease (PD).

One month after the completion of chemotherapy and radiotherapy, oral S-1 (twice daily after meals for 14 consecutive days every 4 weeks) was administered in MC patients (we call this a 2–4 regimen in the current study). The dose of S-1 was determined according to the body surface area (BSA): 40 mg twice a day for BSA < 1.25 m^2^; 50 mg twice a day for 1.25 m^2^⩽ BSA < 1.5 m^2^; and 60 mg twice a day for BSA ⩾1.5 m^2^. MC-S1 was administered for12 or 24 cycles or terminated earlier if there was disease progression, intolerable toxicity, or at the patient’s request.

In patients who developed adverse events during MC, the next chemotherapy cycle was delayed until the toxicities subsided to ⩽ grade 1 for both hematological and nonhematological toxicities. The dose of S1 was modified according to the toxicity profile. In principle, if a patient had a hematological or nonhematological toxicity ⩾ grade 3 or recurrent nonhematological toxicity ⩾ grade 2, one level of dose reduction (DR) was performed, from 60 to 50 mg. If recurrent severe hematological or nonhematological toxicity (⩾ grade 3) persisted despite the DR, one further level of DR was applied, from 50 to 40 mg. If a patient was unable to tolerate 40 mg, the S-1 was permanently withdrawn.

Adetailed description of the IMRT had been previously published [18]. Whenever possible, salvage treatments (including intracavitary brachytherapy, surgery, and chemotherapy) were provided for patients who developed relapse or persistent disease.

### Criteria for toxicity and treatment response

The toxicity of MC was graded using the National Cancer Institute common toxicity criteria (NCI CTC v3.0) [19]. The response to NAC was reassessed no more than 1 week before RT. The short-term response to IMRT was first evaluated on the completion date of RT and was reassessed after 4–6 weeks to confirm. The tumor short-term response to IMRT was defined as the clinically CR, PR, SD and PD using the RECIST guidelines (version 1.1) [20]. These response indicators were based on findings from nasopharyngeal fiberscope and MRI scanning, which were analyzed by two radiologists and confirmed by biopsy if needed.

### Surveillance and statistical analysis

Patients underwent hematologic tests and assessments of clinical symptoms every 2 weeks during S-1 administration. All patients were evaluated every 3 months for the first 2 years after RT, then every 6 months thereafter. Outcomes were evaluated in February 2019. The primary outcomes of interest were overall survival (OS), locoregional failure-free survival (LFFS) and distant metastasis free survival (DMFS). The OS was calculated from the date of diagnosis to the date of death or the last follow-up. LFFS and DMFS were defined as the time from the day of diagnosis to locoregional progression and distant metastasis, respectively. The second endpoint was the toxicity and tolerability of MC.

Survival curves were produced using the Kaplan-Meier estimator method and compared between CRT-MC vs. CRT-non-MC cohorts with the log-rank test. Univariable and multivariable analyses of clinical characteristics, including gender, age, T stage, radiotherapy dose of GTV, regimens and cycles of chemotherapy (including NAC, AC, CC, and MC), potentially associated with survival were performed using the Cox proportional hazards model. Confidence intervals (CIs) represented 95% lower and upper limits.

Patient characteristics and other variables were compared using a Student t-test for continuous variables or a Chi-square test for categorical or ordinal variables. Fisher’s exact test was used when the frequency of a variable was low.

## Results

### Patient characteristics

**Fig. 1:**
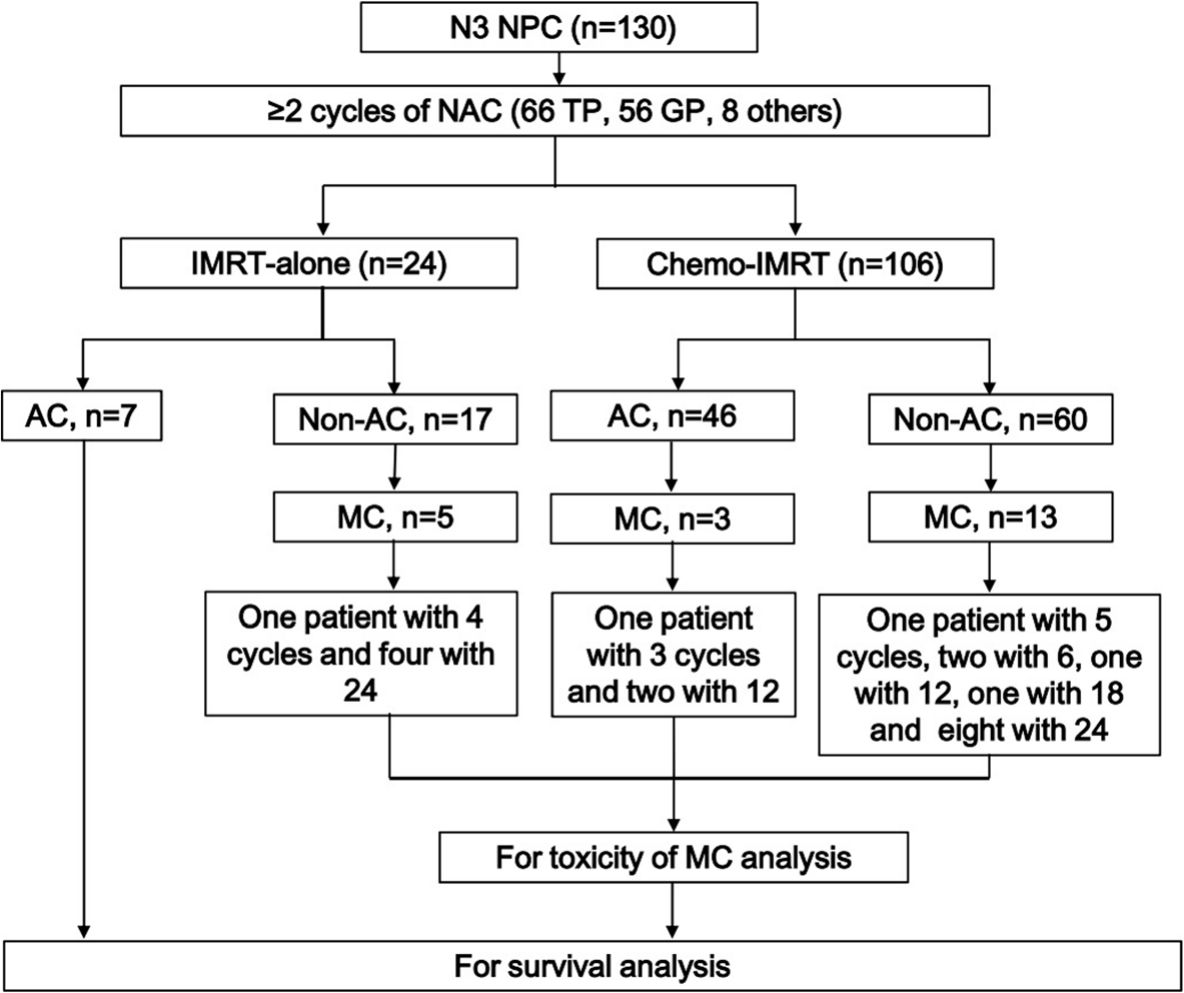
Treatment schedule. Patients with stage N3 nasopharyngeal carcinoma were treated with NAT followed by IMRT alone or CCRT. AC and/or MC as described for certain patients. NAT = neoadjuvant chemotherapy; CCRT = concurrent chemoradiotherapy; IMRT = Intensity Modulated Radiation Therapy; AC = adjuvant chemotherapy; MC = maintenance chemotherapy

A total of 160 pathologically confirmed N3 NPC patients were treated at Fujian Provincial Cancer Hospital (South China) from May 1, 2014 and Dec 31, 2016. We excluded individuals from the study who had received their initial treatment at other hospitals (five patients). We also excluded individuals who had distant metastasis (six patients), those considered ineligible for systemic chemotherapy due to medical conditions (two patients) and those refused treatment (17 patients). The remaining 130 patients were included in the study. According to the guidelines for the treatment of NPC in our hospital, induction chemotherapy combined with concurrent chemoradiotherapy [6] is one of the standard treatment methods for locally advanced NPC. Of the 130 patients, 106 received induction chemotherapy combined with concurrent chemotherapy, three patients had been recruited into a clinical trial and underwent induction chemotherapy combined with radiotherapy alone (NCT02500940). The other 21 patients failed to receive concurrent chemotherapy after induction chemotherapy because of patient tolerance, seven of these patients were described as having adjuvant chemotherapy after radiotherapy (Fig. 1). MC after IMRT was administrated to 21 (16.2%) patients (CRT-MC), and the remaining 109 (83.8%) patients did not get MC (CRT-non-MC).

No statistically significant differences in patient characteristics were identified between the CRT-MC and CRT-non-MC patients in terms of age, gender, pathologictype, T-classification, cycles of CC, cycles of AC, radiation dose to GTV, and short-term treatment response to NAC and RT, except cycles of NAC (Table 1).

**Table 1:** Clinical characteristics of NPC patients with N3 disease

Characteristic	Chemoradiotherapy-non-MC Patients (*n* = 109)	Chemoradiotherapy-MC Patients (*n* = 21)	*p* value
Gender, n (%)			0.101
Male	68 (62.4)	17 (81)	
Female	41 (37.6)	4 (19)	
Age, y			0.989
Median	46	42	
Range	18–65	26–65	
Pathology (World Health Organization)			0.960
Type II	10 (9.2)	2 (9.5)	
Type III	99 (90.8)	19 (90.5)	
Dose (Gy, range)			0.961
GTV	69.96	69.75	
Range	69.3–74.2	69.7–70.95	
T stage, n (%)			0.074
T1	12 (11)	7 (33.3)	
T2	28 (25.7)	6 (28.6)	
T3	36 (33)	4 (19)	
T4	33 (30.3)	4 (19)	
NeoCT (cycles), n (%)			
2	31 (28.4)	0	0.002
3	65 (59.6)	15 (71.4)	
4	10 (9.2)	3 (14.3)	
6	3 (2.8)	3 (14.3)	
ConCT (cycles), n (%)			0.202
0	19 (17.4)	5 (23.8)	
1	21 (19.3)	2 (9.5)	
2	61 (56)	14 (66.7)	
3	8 (7.3)	0	
AdjCT (cycles), n (%)			0.076
0	59 (54.1)	18 (85.7)	
1	35 (32.1)	2 (9.5)	
2	13 (11.9)	1 (4.8)	
3	2 (1.8)	0	
Total chemotherpy (cycles)^a^, n (%)			0.889
Median	5	5	
Range	2–7	3–8	
Response to NeoCT			0.120
CR	1 (0.9)	2 (9.5)	
PR	105 (96.3)	18 (87.7)	
SD	3 (2.8)	1 (4.8)	
Short-term treatment response^b^			0.584
CR	59 (54.1)	10 (47.6)	
PR	50 (45.9)	11 (52.4)	

*Abbreviations*: *MC* Maintenance chemotherapy, *NeoCT* Neoadjuvant chemotherapy, *ConCT* Concurrent chemotherapy, *AdjCT* Adjuvant chemotherapy, *CR* Complete response, *PR* Partial response

^a^Total chemotherapy cycles was caculated as the total cycles of NeoCT, ConCT and AdjCT. ^b^Evaluated 4–6 weeks upon completion of radiotherapy

### Treatment failure pattern and survival analysis of the cohort patients

The median follow-up time was 41 (6–57) months for surviving patients. At the last follow-up, a total of 46 patients had treatment failure, of which nine patients (6.9%) had locoregional failure alone, 32 patients (24.6%) had DM, and there were five cases with both locoregional and distant failure (3.8%). Compared to the CRT-non-MC cohort, the CRT-MC cohort had a higher OS (95.2% [95% CI 86.2–100] vs. 76.3% [95% CI 67.9–84.7], *p* = 0.046) and DMFS (90.5% [95% CI 78–100] vs. 70.3% [95% CI 61.5–79.1], *p* = 0.043) but not LFFS (100% vs. 84.9%, *p* = 0.091) (Table 2).

**Table 2:** Survival rate of NPC patients with N3 disease

Three years	All Patients	Chemoradiotherapy-non-MC Patients (*n* = 109)	Chemoradiotherapy-MC Patients (*n* = 21)	χ^2^	*p* value
OS	79.2%	76.3%	95.2%	2.999	0.046
DMFS	73.6%	70.3%	90.5%	4.111	0.043
LFFS	88.2%	84.9%	100%	2.851	0.091

*Abbreviations*: *MC* Maintenance chemotherapy, *OS* Overall survival, *DMFS* Distant metastasis-survival, *LFFS* Locoregional failure-free survival

**Table 3:** Univariate analysis of prognostic factors

Characteristic	LFFS		DMFS		OS	
	HR(95%CI)	*P* value	HR(95%CI)	*P* value	HR(95%CI)	*P* value
Gender(Male/Female)	0.44 (0.123–1.582)	0.209	0.441 (0.201–0.966)	0.041	0.540 (0.231–1.265)	0.156
Age(y)	0.996 (0.947–1.047)	0.872	1.021 (0.990–1.053)	0.193	1.013 (0.978–1.013)	0.464
Pathology (Type II/III)	0.873 (0.114–6.687)	0.896	1.489 (0.526–4.213)	0.453	2.471 (0.941–6.485)	0.066
GTV Dose (Gy)	0.998 (0.990–1.006)	0.635	1.004 (1.000–1.007)	0.026	1.003 (1.000–1.007)	0.086
T stage(T1/T2/T3/T4)	0.923 (0.561–1.520)	0.754	1.250 (0.905–1.727)	0.176	1.472 (1.000–2.166)	0.050
NeoCT (cycles)	0.982 (0.524–1.840)	0.954	0.716 (0.457–1.121)	0.144	0.598 (0.337–1.060)	0.078
ConCT (cycles)	0.961 (0.509–1.817)	0.904	1.037 (0.698–1.541)	0.856	1.071 (0.681–1.683)	0.767
AdjCT (cycles)	0.937 (0.469–1.870)	0.853	0.983 (0.647–1.494)	0.983	1.207 (0.782–1.862)	0.395
Total chemotherpy (cycles)^a^	0.935 (0.577–1.515)	0.784	0.845 (0.631–1.130)	0.256	0.919 (0.660–1.280)	0.618
Response to NeoCT(CR/PR/SD)	0.856 (0.092–7.936)	0.891	0.510 (0.139–1.878)	0.312	0.503 (0.120–2.105)	0.347
Short-term treatment response^b^(CR/PR)	1.535 (0.533–4.425)	0.427	1.260 (0.661–2.402)	0.483	1.208 (0.583–2.503)	0.611
MC	0.037 (0.000–15.944)	0.287	0.256 (0.062–1.067)	0.061	0.168 (0.023–1.240)	0.080

Abbreviations: GTV, gross tumour volume; NeoCT, neoadjuvant chemotherapy; ConCT, concurrent chemotherapy; AdjCT, adjuvant chemotherapy; CR, complete response; PR, partial response; MC, maintenance chemotherapy; HR = Hazard ratio, CI=Confidence interval

^a^Total chemotherapy cycles was caculated as the total cycles of NeoCT, ConCT and AdjCT. ^b^Evaluated 4–6 weeks upon completion of radiotherapy

Univariate and multivariate analyses revealed that MC (HR, 0.217; 95% CI 0.052–0.906; *p* = 0.036) and gender (HR, 0.389; 95% CI, 0.177–0.854; *p* = 0.019), were the only two independent factors that significantly influenced DMFS (Tables 3 and 4). The T stage (HR, 1.472; 95% CI, 1–2.166; *p* = 0.05) was the only factor to significantly impact OS (Table 4). No independent prognostic factor was found for LFFS. The cycles of NAC failed to show significant positive trends for all endpoints.

**Table 4:** Independent prognostic factors by multivariate analyses for patients with N3 stage

Endpoint	Factor	*P* value	HR(95%CI)
Distant failure	MC	0.036	0.217 (0.052–0.906)
	Gender	0.019	0.389 (0.177–0.854)
Death	T stage	0.05	1.472 (1–2.166)

*HR* Hazard ratio, *CI* onfidence interval

### Toxicities and efficacy of MC

A total of 366 cycles, with a median 24 (3–24) cycles, of MC were administrated to the CRT-MC group of patients. Of which, 12 patients received 24 cycles, and another three patients received 12 cycles and one received 18 cycles of MC-S1. Three patients discontinued MC at 5–6 cycles of MC due to repeated grade 2 leukopenia. Two patients discontinued MC without giving any reasons at the end of the third and fourth cycle of MC (Fig. 1).

No grade 3/4 toxicity or treatment-related deaths occurred during MC. The most common toxicity of MC was grade 1–2 skin hyperpigmentation, leukopenia, fatigue and neutropenia. Gastrointestinal toxicities, which are the greatest limitation to chemotherapy with S-1, were very minimal with grade 1 anorexia and/or nausea in the current study with S-1 as the MC regimen (Table 5).

**Table 5:** Adverse events during maintenance chemotherapy (*n* = 21)

Toxicity	Grade 1		Grade 2	
	No.	%	No.	%
Any of the following	8	38.1	11	52.4
Neutropenia	7	33.3	3	14.3
Leucopenia	6	28.6	5	23.8
Anaemia	2	9.5	1	4.8
Thrombocytopenia	0	0	2	9.5
Mucositis oral	1	4.8	0	0
Vomiting	2	9.5	0	0
Nausea	6	28.6	0	0
Anorexia	7	33.3	0	0
Diarrhea	3	14.3	1	4.8
Skin hyperpigmentatio	7	33.3	5	23.8
Hepatoxicity	3	14.3	0	0
Fatigue	11	52.4	0	0

**Fig. 2:**
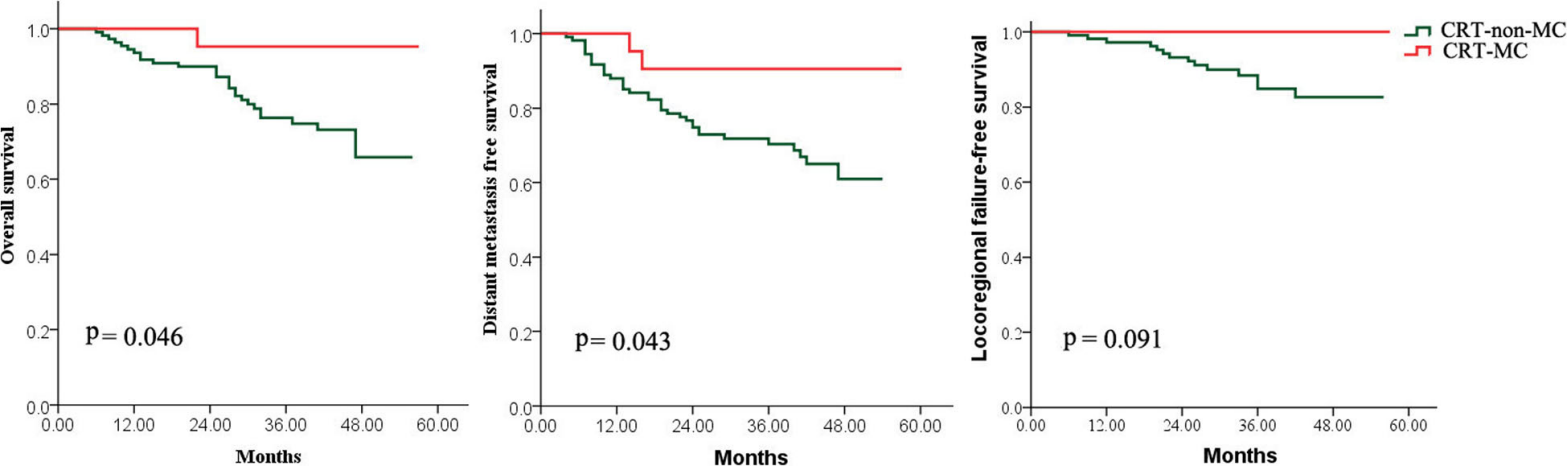
Kaplan–Meier estimates of patients treated with chemoradiotherapy with or without oral maintenance chemotherapy (CRT-MC vs CRT-non-MC)

At the last follow-up, 19 surviving patients remained disease free and two patients who refused MC at the end of third and fourth cycle of MC developed bone metastases 4–6 months after MC. Compared to the CRT-non-MC patients, CRT-MC patients benefit significantly in OS (*p* = 0.046) and DMFS (*p* = 0.043) but not in LFFS (*p* = 0.091) (Fig. 2).

## Discussion

Hong et al. had reported that MC with weekly 5-fluorouracil, leucovorin was effective and had minimal side-effects for treating metastatic NPC [11]. However, the efficacy of MC in patients treated with definitive treatment has rarely been reported. There have been several reports that argued that AC following definitive CRT could not improve the survival of NPC patients [21], questioning the effectiveness of MC in NPC following definitive RT. Fortunately, the results of the present polit study are quite encouraging albeit its small sample size and retrospective non-randomized nature. Compared with non-MC patients, MC-S1 achieved superior survival benefits for both OS and DMFS. This result suggests that MC-S1 might have a greater role in treating N3-NPC patients and deserves further clinical study.

Besides efficacy, the toxicity and tolerability are important factors in selecting a regimen for making MC decision. In the present study, a total of 366 cycles of MC were delivered to 21 patients with a median of 24 cycles (3–34 cycles). Besides skin hyperpigmentation, hematological toxicities were the most common in response to MC. The most sever toxicity were grade 2 leukopenia which were preventable with granulocyte colony stimulating factor treatment. Only three patients were required to reduce the dose of MC and were unwilling to continue with their therapy due to the grade 2 leukopenia. The gastrointestinal toxicities and stomatitis are other commonly reported toxicities of MC with S-1 [5]. However, as demonstrated in this study, only one patient (4.5%) encountered grade II gastrointestinal toxicities and no nephrotoxicity was observed. All these mild toxicities indicated that the S-1 was an appropriate and tolerable regimen for MC.

Currently, the best dose intensity and duration of MC-S1 for N3-NPC is not determined. Although we were unable to analyze the impact of various MC cycles on prognosis due to the limited number of patients enrolled in this study, a retrospective study of MC-S1 reported by Misato Hirai [22] for advanced squamous cell carcinoma of the head and neck (SCCHN) may serve as a reference. In that study, the clinical records of 89 patients with SCCHN who underwent adjuvant chemotherapy with S-1 were investigated. They found that S-1 administration for periods of 24 months or longer showed significantly lower hazard ratios (HRs) than administration for 0–12 months. Furthermore, our previous studies had found that most (73%) of metastasis occurred within 2-years after RT with the probability of metastasis after 2-years less than 25% [23]. Therefore, if we could control metastasis within the first 2-years after CRT by means of MC, there is about a 70% probability of DMFS with improved OS. Based on these conclusions, we believe that at least one year and at best a 2-year duration of MC-S1 may be an effective MC to decrease the patient’s DMFS. However, one notable finding of the current study was that both patients who had ≤4 cycles of MC developed distant metastasis a short time after MC suspension, whereas all 19 patients who received > 5 cycles of MC had disease free survival. The results suggest that at least 5 cycles of MC with S-1 were needed to achieve a survival improvement by MC. Certainly, in addition to the minimum required cycles of MC, the maximum number of cycles of MC are also of concern to clinicians. Unfortunately, because MC is usually adopted as a palliative treatment for patients with advanced cancer, the appropriate maximum number of cycles of MC has not been established, even in cancers like lung and rectal with skilled application of MC. In this study, as of the last follow-up date, no significant difference of DMFS were observed among the 19 patients with > 5 cycles, although they had various numbers of MC cycles. It seemed as if five cycles of MC were appropriate, however, because of the limitation of the number of enrolled cases, especially patients who accepted five cycles of MC, we will require more research.

Several clinical studies had been conducted on the different strategies of dosing schedules and intensity of chemotherapy using S-1, including four consecutive weeks every 6 weeks (regimen4–6) [5, 24] 2–3 weeks [14] and 2–4 weeks [16]. Up to the present, few studies have been conducted to optimize the treatment schedule for S-1. The 2–4 week [16] regimen had been reported to have mild toxicity and been chosen for the present study considering the poor compliance of adjuvant chemotherapy after radiotherapy for NPC patients [6]. The current MC regimen had resulted in superior survival benefit and mild toxicity and seemed to be a good selection for N3 NPC patients.

There are certain limitations to the present study, such as the retrospective design, disunified chemotherapy regimens, the unbalanced number of NAC cycles between the two groups, the small sample enrolled and the short surveillance time. Due to these limitations, the results of our investigation must be interpreted with caution.

## Conclusions

MC-S1 in N3-NPC following IMRT achieved superior survival to the non-MC group. The toxicities of MC were mild and tolerable. The retrospective nature of the current study serves as a limitation; thus, further clinical trials are required to evaluate the efficacy of MC-S1 in N3-NPC patients.

## Data Availability

All data generated or analyzed during this study are included in this published article.

## Abbreviations

AC: Adjuvant chemotherapy
BSA: Body surfacearea
CC: Concurrent chemotherapy
CCRT: Concurrent chemoradiotherapy
CR: Complete response
CRT: Chemoradiotherapy
CRT-MC: Chemoradiotherapy with maintenance chemotherapy
CRT-non-MC: Chemoradiotherapy without maintenance chemotherapy
DM: Distant metastasis
DMFS: Distant metastasis free survival
IMRT: Intensity-modulated radiotherapy
LFFS: Locoregional failure-free survival
MC: Maintenance chemotherapy
MC-S1: Maintenance chemotherapy using S-1
NAC: Neoadjuvant chemotherapy
NPC: Nasopharyngeal carcinoma
OS: Overall survival
PD: Progression of disease
PR: Partial response
RT: Radiotherapy
SD: Stable disease
